# Boosting Electrochemical Nitrogen Reduction Performance over Binuclear Mo Atoms on N-Doped Nanoporous Graphene: A Theoretical Investigation

**DOI:** 10.3390/molecules24091777

**Published:** 2019-05-08

**Authors:** Ruijie Guo, Min Hu, Weiqing Zhang, Jia He

**Affiliations:** Tianjin Key Lab of Advanced Functional Porous Materials, Institute for New Energy Materials and Low-Carbon Technologies, School of Materials Science and Engineering, Tianjin University of Technology, Tianjin 300384, China; grj_0607@163.com (R.G.); humin960910@163.com (M.H.); zhangweiqing@tjut.edu.cn (W.Z.)

**Keywords:** nitrogen reduction reaction, single atom catalyst, binuclear atom catalyst, first principle calculation

## Abstract

Exploration of efficient catalysts is a priority for the electrochemical nitrogen reduction reaction (NRR) in order to receive a high product yield rate and faradaic efficiency of NH_3_, under ambient conditions. In the present contribution, the binding free energy of N_2_, NNH, and NH_2_ were used as descriptors to screen the potential NRR electrocatalyst among different single or binuclear transition metal atoms on N-doped nanoporous graphene. Results showed that the binuclear Mo catalyst might exhibit the highest catalytic activity. Further free energy profiles confirmed that binuclear Mo catalysts possess the lowest potential determining step (hydrogenation of NH_2_* to NH_3_). The improved activities could be ascribed to a down-shift of the density of states for Mo atoms. This investigation could contribute to the design of a highly active NRR electrocatalyst.

## 1. Introduction

Nitrogen fixation is one of the most important process from the perspectives of agriculture and human development [1,2,3,4,5,6]. Traditional Haber–Bosch process consumes plenty of energy, due to the difficulty of direct nitrogen-nitrogen triple bond cleavage reaction [7]. Electrocatalytic N_2_ reduction reaction (NRR) holds a great promise for realizing NH_3_ production, as it can decrease the barrier height of breakage for a nitrogen-nitrogen bond, to a certain degree [8,9,10,11,12,13]. Different kinds of electrocatalysts, such as single atom catalysts (SACs) [14,15,16,17,18,19,20], have been developed for boosting the nitrogen reduction performance. For the majority of catalysis, the intrinsic activities of SAC are very high, compared to the state-of-art catalysts. Zhao et al. have shown that, typically, a single Mo atom supported on a defective boron nitride monolayer is demonstrated to be an efficient electrocatalyst for nitrogen fixation, with a very low potential determining step (PDS) value, via the enzymatic pathway [21]. As the more widely used two-dimensional catalysts, single Mo and Fe atoms supported by N-doped nanoporous graphene (Mo- or Fe-N-C) have also been revealed to exhibit a good catalytic activity for NRR [22,23]. However, in general, the NRR strategy is still hindered by its high potential barrier, leading to an extremely low production rate and Faradaic efficiency of NH_3_.

Previous investigations have indicated that an excellent NRR electrocatalyst should have a moderate adsorption energy for N-related species [24,25]. In most cases, such as single Mo and Fe atoms supported by N-doped nanoporous graphene, SACs might show stronger or weaker interaction energy towards the reactant. Therefore, the electronic structure needs to be adjusted to get a proper binding energy. It has previously been reported that changing one atom in the ultrafine cluster might largely alter the electronic structure and drastically change its catalytic properties [26,27]. Such atom-dependent catalytic behaviors have been successfully demonstrated by the model catalysts of mass-selected metal clusters. Thus, atomically precise ultrafine metal clusters, such as dimers or trimer, on high-surface area supports, might improve the intrinsic activities of a catalyst, by adjusting the adsorption energy for N-related species.

Recently, Lu et al. have showed that Pt_2_ dimers can be fabricated with a bottom-up approach on graphene, using the atomic layer deposition [28]. In the hydrolytic dehydrogenation of ammonia borane, Pt_2_ dimers were found to exhibit a higher specific rate of 2800 mol_H2_ mol_Pt_^−1^ min^−1^, which was ~17-fold higher than the graphene-supported Pt single atoms. Li et al. developed a host–guest strategy to fabricate electrocatalysts with Fe-Co dual sites embedded on N-doped nanoporous graphene, and further showed an excellent oxygen reduction activity in the acidic electrolyte [29]. Li et al. proposed a new strategy that anchored an Fe_3_ or Co_3_Rh cluster on the oxide surfaces, as a heterogeneous catalyst for ammonia synthesis, from the first-principles study and microkinetic analysis [12,30]. The calculated turnover frequency is comparable to the Ru catalyst. However, to our knowledge, no binuclear atoms catalysts (BAC) on 2D materials has been reported for electrochemical NRR on experimental and theoretical fronts. Even to date, the intrinsic reaction mechanism still remains elusive.

Therefore, in this work, the adsorption energy of N_2_, NNH, and NH_2_ obtained by the density functional theory (DFT) were utilized to screen the electrochemical NRR catalyst for different SAC and BAC, indicating that an Mo BAC is potential excellent N_2_ electrocatalyst. Then, free energy differences along three possible pathways have confirmed that Mo BAC possess a low potential determining step than an SAC, due to the down-shift of states for the Mo atoms relative to the Fermi energy. Additionally, the stabilities of different single or binuclear transition metal atoms on N-doped graphene were investigated, demonstrating that most transition metal BAC were more stable than those of SAC. The conclusions in this work would provide an efficient method for exploring the NRR catalysts.

## 2. Results and Discussion

### 2.1. Stability for Various Mono- and Binuclear N-C Catalysts

**Figure 1:**
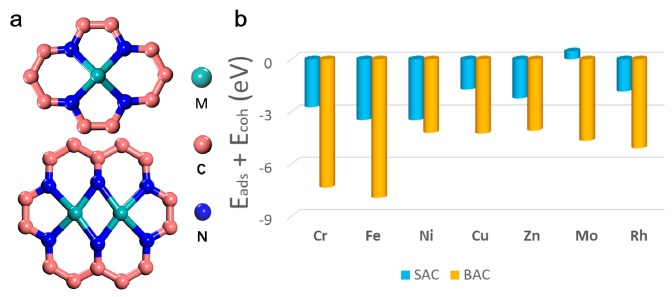
(**a**) Structures of single and binuclear atoms catalysts, and (**b**) calculated formation energy of different mono- and binuclear N-C catalysts.

It is well-known that the duration of SAC or BAC is a key challenge in their applications that is yet to be solved [31]. As shown in Figure 1, the stability of different single or binuclear transition metal atoms on N-doped graphene was tested via their formation energies. Figure 1a shows the structural model of mono- and binuclear catalysts. Different possible structures of SAC and BAC, such as Mo-based catalysts, was explored, as depicted in Figure S1 in the Supplementary Materials (SM). Results showed that the configurations in Figure 1a were more favorable and, thus, stable (Table S1). Figure 1b and Table S2 shows the formation energies of various mono- and binuclear catalysts for different transition metal anchored on the N-doped graphene (N-C). The stabilities at the given potentials for the different metal centers, were also collected, and is shown in Table S3. It can be seen that the majority of SAC and BAC had a good stability, even under harsh electrochemical conditions. Results indicate that the order of stability for mononuclear N-C was: Ni > Fe > Cr > Zn > Rh > Cu > Mo. The order of stability for the binuclear N-C was: Fe > Cr > Rh > Mo > Cu > Ni > Zn. An interesting result was that, for certain metal-doped monolayer, formation of a second metal center was energetically favorable. Typically, the stability of the BAC for Mo and Ru is much higher than that of SAC, which further confirmed the rationality of our catalyst design.

### 2.2. Screen of Potential NRR Electrocatalyst Combining Different Descriptors

**Figure 2:**
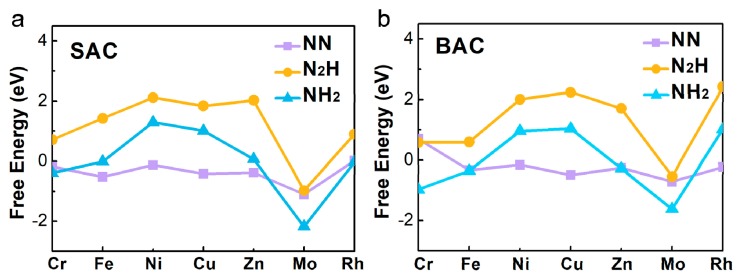
The calculated Gibbs free energies of N_2_, NNH, and NH_2_ species on various (**a**) mono- and (**b**) binuclear N-C catalysts.

Based on the three screeners of eligible electrocatalyst for the NRR proposed by the previous studies [21,24,25], we explored a series of metal atoms, supported by defective N-C nanosheets, by calculating the adsorption free energy (∆G) of N_2_, NNH, and NH_2_ (Figure 2 and Table S4). As shown in Figure 2a, it indicated that Fe- and Mo- mononuclear catalyst had a lower ∆G for N_2_ adsorption (∆G_Fe_ = −0.52 eV, ∆G_Mo_ = −1.10 eV). For the binuclear catalysts, Fe and Mo had a lower ∆G (∆G_Fe_ = −0.33 eV, ∆G_Mo_ = −0.71 eV) than the other metals, for the adsorption of N_2_. It could be inferred that these two metal-doped nanosheets were more favorable NRR electrocatalysts because of the strong N_2_ activation. Figure 2 also shows the adsorption free energies of NNH and NH_2_ on the mononuclear and binuclear catalysts. We found that the properties of mononuclear and binuclear catalysts for Fe and Mo were interesting. As shown in Figure 2a, ∆G_NNH_ on Fe-N-C was calculated to be as high as 1.43 eV, and ∆G_NH2_ on Mo-N-C was as low as −2.18 eV, showing that for Fe and Mo, anchored SAC was not suitable for NRR. However, in the BAC catalysts, ∆G_NNH_ on Fe_2_-N-C was 0.60 eV, which was much smaller than that on the Fe-N-C. Binding free energy of NH_2_ on Mo_2_-N-C was −1.62 eV, which was also smaller than that on Mo-N-C. More importantly, Mo_2_-N-C possessed the lowest difference value between NNH and NH_2_, than the others. Therefore, it could be inferred that Mo_2_-N-C might have a better performance for NRR than Mo-N-C and the other SAC. Combined with the above analyses results, our interest on further exploration of Mo-anchored catalysts, was aroused.

### 2.3. Mo- and Mo_2_-N-C Monolayer for NRR

**Figure 3:**
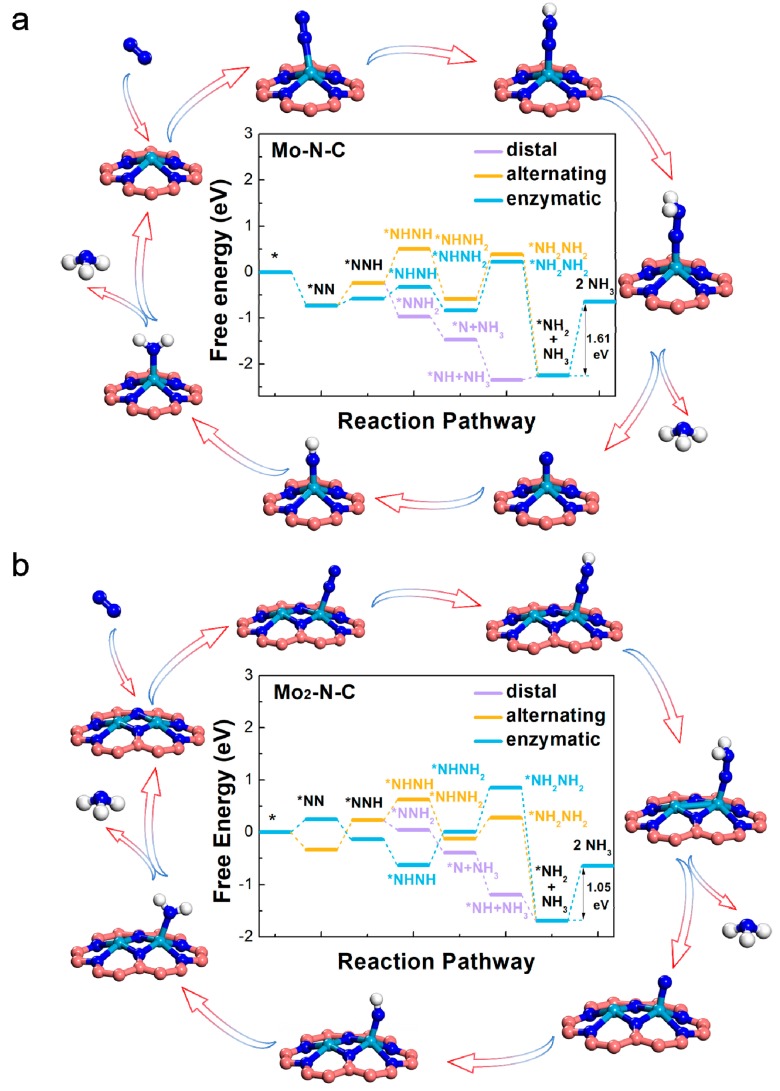
Free-energy diagrams for the nitrogen reduction reaction (NRR) on the Mo-N-C (**a**) and Mo_2_-N-C (**b**) catalysts.

To assess the ability of Mo-N-C and Mo_2_-N-C monolayer as an electrocatalyst to reduce activated N_2_ to NH_3_, we explored four possible NRR pathways, including distal, alternating, enzymatic, and dissociative mechanisms. Figure 3, and Tables S5 and S6 summarize the atomic configurations and corresponding free energy changes of each elementary steps, corresponding to the different paths. Energy zero was defined as the energy of pure SAC or BAC, and the free N_2_ molecule. For Mo-N-C, the ΔG values of PDS, through distal, alternating, and enzymatic pathways were calculated to be 1.61 eV, corresponding to the hydrogenation of NH_2_* to NH_3_. For Mo_2_-N-C, hydrogenation of NH_2_* to NH_3_ was also calculated to be PDS (1.05 eV), through the distal, alternating, and enzymatic pathways. By further comparing the ΔG values of PDS, it could be inferred that the catalytic performance of Mo_2_-N-C was better than that of Mo-N-C. Single and binuclear Mo atoms on N-doped graphene also exhibited an excellent NRR activity, in comparison to other electrocatalysts, especially for the carbon-based materials with the same PDS (Table S7).

**Figure 4:**
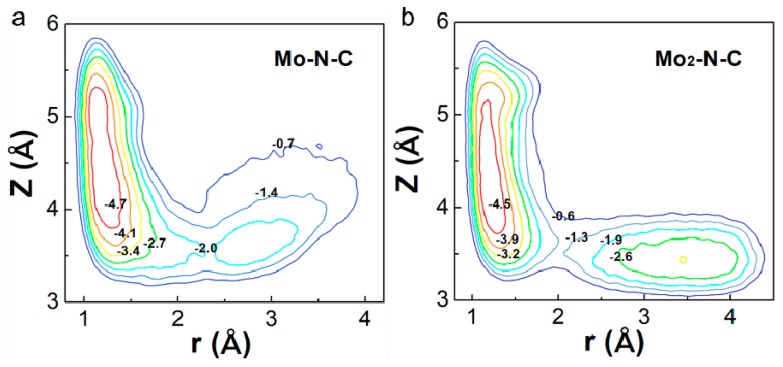
The potential energy surfaces of N_2_ dissociation on Mo (**a**) mono- and (**b**) binuclear N-C catalysts.

Generally, a dissociative pathway is difficult to achieve because of the extremely stable nitrogen–nitrogen triple bond. However, Mo_2_-N-C might facilitate a direct dissociation of N_2_, due to its unique electronic structure and catalytic properties. Thus, the potential energy surface (PES) as a function of the molecular coordinates r and z (see Figure 4) were calculated for Mo-N-C and Mo_2_-N-C. PES were obtained using DFT–molecular dynamics (MD) simulations. To estimate the free energy barriers for the dissociative reaction, we employed metadynamics, which allowed to sample the free energy landscape spanned by the two collective variables whose combination was able to describe the mechanism under study. The method has been successfully used to investigate the dissociative pathway of different small molecules on the catalyst surface [32]. For Mo-N-C, it could be seen that only one remarkable minimum was obtained, demonstrating that N_2_ was hardly directly decomposed. As for Mo_2_-N-C, another minimum was observed, in which the N_2_ had been broken into two N atoms absorbed on two Mo atoms. Results showed that binuclear Mo N-C nanosheet were the more favorable NRR electrocatalyst due to the strong N_2_ activation.

**Figure 5:**
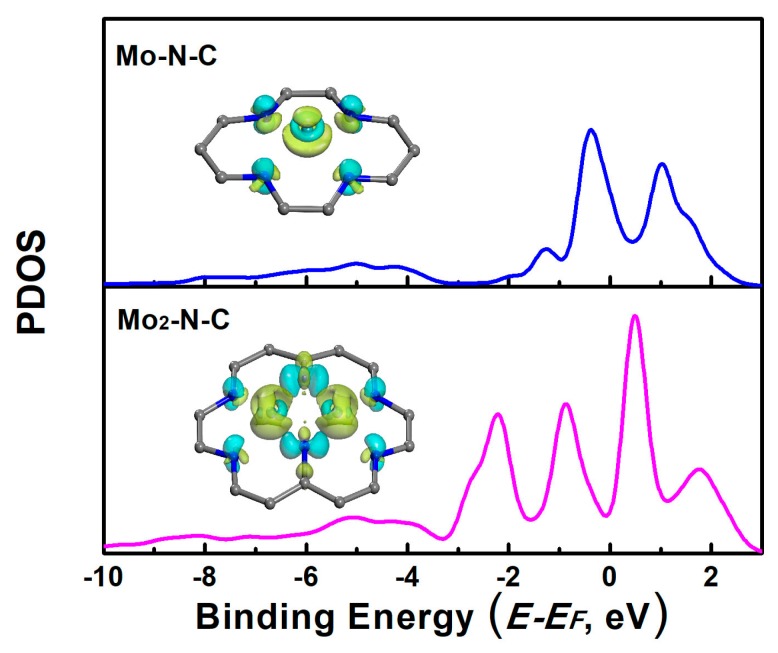
Projected density of the states (PDOS) of Mo atoms on mono- and binuclear N-C catalysts. Inset: the charge density difference of the Mo adsorption configuration on Mo-N-C and Mo_2_-N-C catalysts (the cyan and yellow colors represent charge accumulation and depletion in the space, respectively).

In order to further investigate the factors influencing the performance of the catalyst, we further calculated the projected density of state (PDOS) of Mo-N-C and Mo_2_-N-C. As shown in Figure 5, the PDOS of Mo on the Mo-N-C had a peak on the left sides of the Fermi level, and the peak was very close to the Fermi level. It could be inferred that the Mo-N-C would have an excessive interaction with the reactants. The excessive adsorption caused it to be difficult to get detached from the catalyst surface. As for the Mo_2_-N-C, the peak width of Mo was not sharp, and the highest peak was far away from the Fermi level. This indicated that the 4d orbital of Mo was hybridized, due to the interactions with the carrier. The shift down of the peak of the PDOS resulted in the moderate binding strength of the reactants. It could also be deduced from the charge density difference of the Mo-embedded configuration on the monolayer (insets of Figure 5). For the Mo-N-C, electrons, both assumption and depletion towards the adsorbate appeared, while for the Mo_2_-N-C, electrons depletion was dominant, leading to a decrease of the binding ability.

Owing to the oxophilicity of the metal atom, *OH could be formed spontaneously in an aqueous environment, at applied potentials. Previous investigation has demonstrated that the pre-adsorbed OH on SAC or BAC acted as a modifying ligand to promote electrochemical activity [27]. In order to clarify this effect, PDOS of metal atoms on Mo-N-C and Mo_2_-N-C with pre-adsorbed OH were, thus, analyzed preliminarily (Figures S2 and S3). It could be inferred that the pre-adsorbed OH induced the shift down of peak of PDOS, under the Fermi Energy for Mo-N-C (from −0.38 eV to −0.92 eV) and Mo_2_-N-C (from −0.88 eV to −2.36 eV), leading to a weaker binding strength of the reactants, such as NH_2_*. Free energy diagrams of NRR on Mo-N-C and Mo_2_-N-C have shown that hydrogenation of NH_2_* to NH_3_ was a PDS, along different pathways. Therefore, Mo-N-C and Mo_2_-N-C with pre-adsorbed *OH might also exhibit excellent NRR activity. Moreover, in order to preliminarily explore the activity on a clean Mo surface, PDOS of the metal atoms on Mo (001) were calculated (Figure S4). It can be seen that the peak of PDOS under Fermi Energy was located at −0.37 eV, which was closer to the Fermi Energy, compared to those of Mo-N-C (−0.38 eV) and Mo_2_-N-C (−0.88 eV). Thus, the binding ability of NH_2_* might be stronger on the Mo surface and the NRR activity of Mo surface should be less favorable than those of Mo-N-C and Mo_2_-N-C.

### 2.4. Hydrogen Evolution Reaction

**Figure 6:**
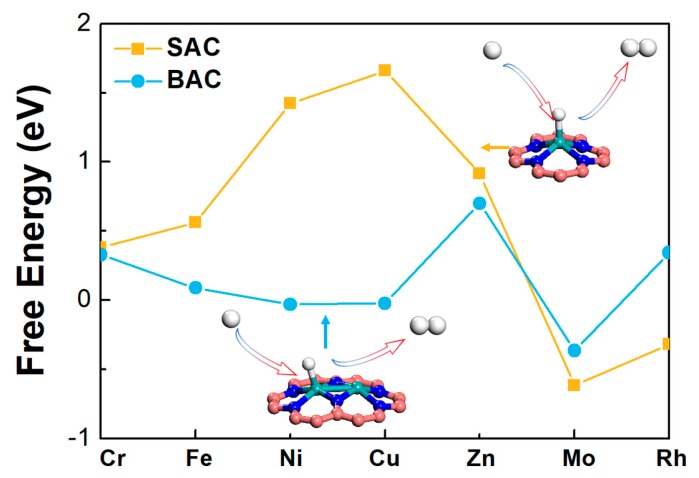
The calculated Gibbs free energy changes of HER on mono- and binuclear N-C catalysts.

It is important to consider the unwanted hydrogen evolution reaction, since most current densities observed in the experiments for the NRR were hydrogen evolution reaction (HER). Therefore, the selectivity of the catalyst for NRR was evaluated by calculating the reaction free energy of HER (Figure 6) [33,34]. Results indicated that the order of HER activities for mononuclear N-C was: Rh > Cr > Fe > Mo > Zn > Ni > Cu. The order of HER activities for the binuclear N-C was: Cu > Ni > Fe > Cr > Rh > Mo > Zn. It could be seen that, for most BAC, the HER activities were also improved, accompanied by the enhancement of NRR. However, the free energy change was much smaller, especially for the Mo-based catalyst. The improved activities could also be ascribed to the down-shift of the density of states for the Mo atom. Furthermore, electrode potentials of different reaction were estimated, based on the potential determining step. Under this applied potential, all steps involving the proton-electron pairs would go downhill, on the free energy diagram, using a computational hydrogen electrode scheme. It could be seen that the applied potentials of HER for Mo-N-C (U = −0.62 V) and Mo_2_-N-C (U = −0.37 V) was smaller than those of NRR for Mo-N-C (U = −1.61 V) and Mo_2_-N-C (U = −1.05 V) (Figure S5), demonstrating that HER would go in parallel with NRR. Thus, most current densities observed in the experiments for the NRR were HER. However, for the Mo-based SAC and BAC, the free energy change in HER was smaller than that in NRR, therefore, the overall activity of NRR for Mo_2_-N-C should be improved.

## 3. Models and Methods

All computations were implemented by means of spin-polarized density functional theory (DFT) methods, using the DMol^3^ code [35]. The Perdew, Burke, and Ernzerhof (PBE) exchange-correlation functional was employed [36] within a generalized gradient approximation (GGA). The double numerical plus polarization (DNP) was chosen as the base set [37]. Core treatment was adopted as the Effective Core Potentials, to conduct metal relativistic effect. Self-consistent field (SCF) calculations were performed with a convergence criterion of 2*10^−5^ Ha on the total energy and electronic computations. The maximum force and displacement for the geometric optimization were 0.004 Ha/Å and 0.005 Å. We chose a real-space global orbital cutoff radius as high as 4.5 Å. A smearing of 0.005 Ha to the orbital occupation was applied. Using a 5 × 5 × 1 Monkhorst-Pack, grid k-points were employed for the geometric optimization. Structural optimization was carried out without any constraints.

To model the defective N-C monolayer, we first built a supercell containing 60 carbon atoms with a vacuum of at least 15 Å in the z-direction, and then removed six carbon atoms to provide an anchoring site for the single transition metal atom, or removed ten carbon atoms for the binuclear transition metal atoms. Based on the approach described in [38], formation energy was calculated, first, by the adsorption energy of metal atoms over N-doped graphene, and then further correlated with the experimentally obtained cohesive energy. Adsorption energy was calculated according to the equation: ∆E = E_M-N-C_ − (*m*E_M_ + E_NC_), where E_M-N-C_ was the total energy of M-N-C sheet; E_NC_ was the energy of the N-doped graphene without the metal atoms; E_M_ was the energy of the isolated metal atom and *m* was the number of metal atoms in the system. The Gibbs free energy change (∆G) of every basic step involving the electron/proton transferred, was calculated by using the computational hydrogen electrode (CHE) model presented by Nørskov [39]. Based on this method, the ∆G value could be determined as follows: ∆G = ∆E + ∆ZPE − T∆S + ∫C_p_dT, where ∆E was the electronic energy difference calculated from DFT, ∆ZPE was the change in zero-point energies, here T was the ambient temperature, C_p_ was the heat capacity, and ∆S was the entropy change. The thermodynamic properties of molecules in the gas- and adsorbed-phase were obtained through the vibrational frequencies.

Potential energy surfaces (PES) were obtained using semi-empirical PM6 DFT [40] molecular dynamics (MD) simulations, implemented with the open-source CP2K/QUICKSTEP code [41,42]. The choice of PM6, due to its less computational cost, allowed us to explore the long dynamics trajectories, and to recognize the intermediate states of the reaction. To sample the catalyst surface in MD calculations, NVT simulations were performed over 0.5 ns at 500 K, using time-steps of 1 fs.

## 4. Conclusions

In summary, through the spin-polarized DFT calculation and molecular dynamics simulation, we systematically studied the potential of doping the excess metal atoms (Cr, Ni, Fe, Cu, Zn, Mo, and Rh) on the defective N-C catalyst, for NRR. Our calculations showed that the catalytic activity of Mo-doped dual-core catalysts was much higher than that of the mononuclear and other catalysts. Hydrogenation of NH_2_* to NH_3_ was calculated to be a PDS, along the different pathways. The distal pathway was preferred for the Mo_2_-N-C in the NRR reaction. The boosted NRR activity was mainly due to the fact that the DOS of the Mo atom moved far away from the Fermi Energy. The electronic structure was supposed to be adjusted to get the proper binding energy to reactants. This study provided new insights into the development of robust electrocatalysts for the high-efficiency conversion of N_2_ into valuable chemicals.

## Supplementary Materials

The following are available online, Figure S1: Different possible structures of SAC and BAC for the Mo atoms; Figure S2: PDOS of Mo atoms on OH pre-adsorbed Mo-N-C catalysts; Figure S3: PDOS of Mo atoms on OH pre-adsorbed Mo_2_-N-C catalysts; Figure S4: PDOS of Mo atoms on Mo (001) catalysts; Figure S5: Free-energy diagrams for the NRR on Mo-N-C (a) and Mo_2_-N-C (b) catalysts under different potentials via distal pathway; Table S1: The adsorption energy (E_ads_, eV) and the cohesive energy (E_coh_, eV) of the corresponding SAC and BAC for the Mo atoms in Figure S1; Table S2: The adsorption energy (E_ads_, eV) and the cohesive energy (E_coh_, eV) of the different transition metals doped on N-C nanosheets; Table S3: Calculated values of $E_{M^{z+}/\text{M-N-C}}^{o}$(V); Table S4: Adsorption energy (eV) of N_2_, NNH, and NH_2_ intermediates on the different N-C monolayers; Table S5: Atomic configurations and corresponding adsorption energy and free energy correction of each elementary steps along the different pathways for Mo-N-C; Table S6: Atomic configurations and corresponding adsorption energy and free energy correction of each elementary steps, along the different pathways for Mo_2_-N-C; Table S7: Different two-dimensional NRR electrocatalysts reported in the literature.
