# Supplementary material for: Boosting Electrochemical Nitrogen Reduction Performance over Binuclear Mo Atoms on N-Doped Nanoporous Graphene: A Theoretical Investigation
Source: Molecules. 2019 May 8;24(9):1777. doi: 10.3390/molecules24091777 (PMC6539356; doi:10.3390/molecules24091777)
Supplement: Supplementary file 1 [file molecules-24-01777-s001.pdf]

**Supplementary Material**

# Boosting Electrochemical Nitrogen Reduction Performance over Binuclear Mo Atoms on N-doped Nanoporous Graphene: A Theoretical Investigation

Ruijie Guo, Min Hu, Weiqing Zhang, Jia He*

*Tianjin Key Lab of Advanced Functional Porous Materials, Institute for New Energy Materials and Low-Carbon Technologies, School of Materials Science and Engineering, Tianjin University of Technology, Tianjin 300384, China.*

**E-mail: hejia@tjut.edu.cn*

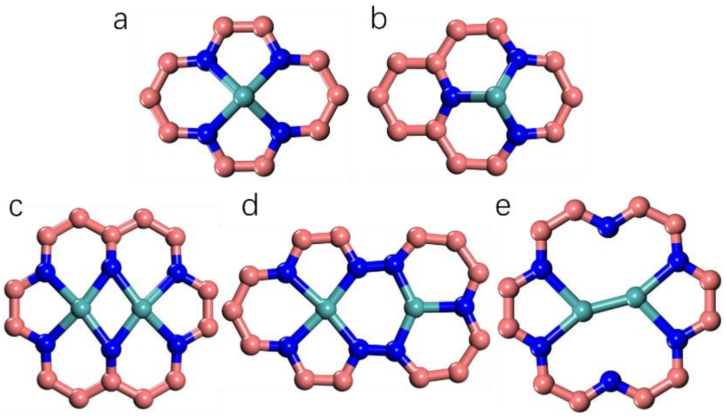


**Figure S1.** Different possible structures of SAC and BAC for the Mo atoms.

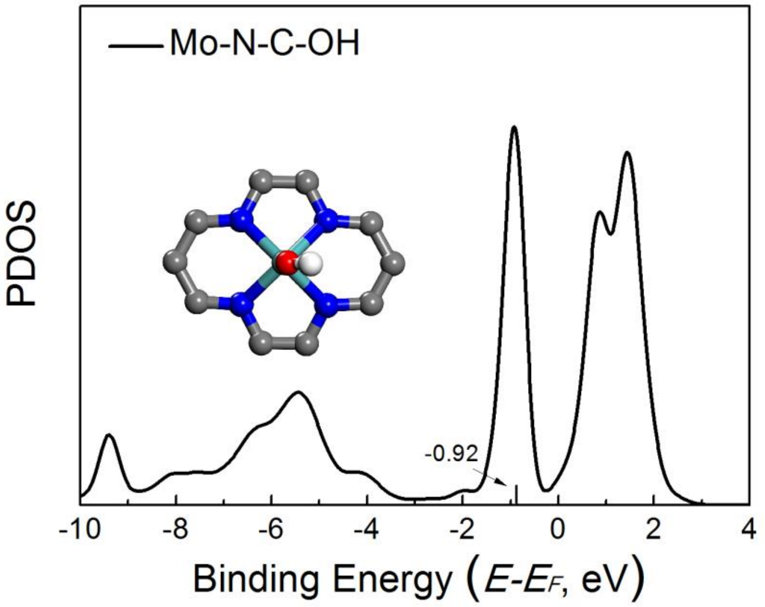


**Figure S2.** PDOS of Mo atoms on OH pre-adsorbed Mo-N-C catalysts.

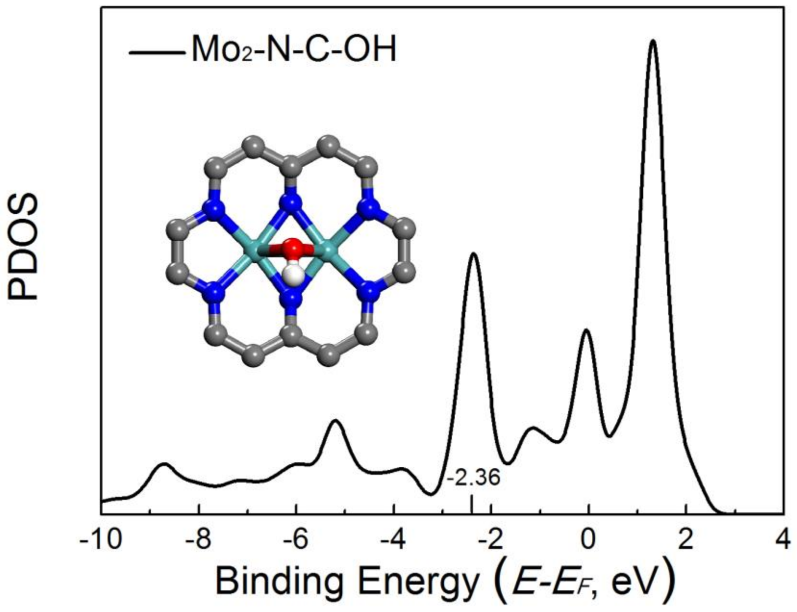


**Figure S3.** PDOS of Mo atoms on OH pre-adsorbed $Mo_2$-N-C catalysts.

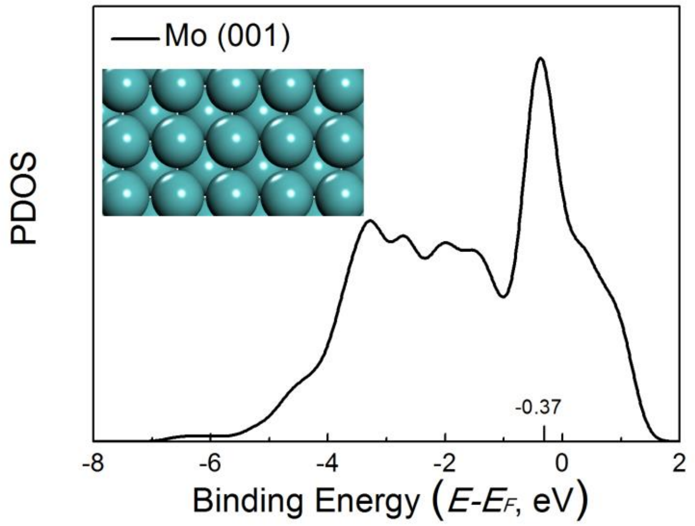


**Figure S4.** PDOS of Mo atoms on Mo (001) catalysts.

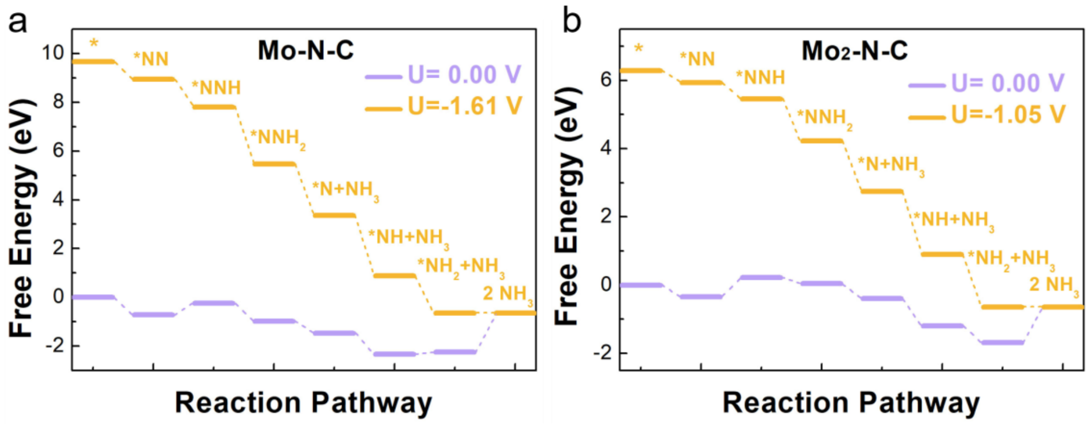


**Figure S5.** Free-energy diagrams for the NRR on Mo-N-C (**a**) and $Mo_2$-N-C (**b**) catalysts under different potentials via distal pathway.

**Table S1.** The adsorption energy ($E_{ads}$, eV) and the cohesive energy ($E_{coh}$, eV) of the corresponding SAC and BAC for the Mo atoms in Figure S1.

| | $E_{ads}$ | $E_{ads} + E_{coh}$ |
|---|---|---|
| a | -6.37 | 0.45 |
| b | 2.72 | 9.54 |
| c | -18.32 | -4.68 |
| d | 7.53 | 21.17 |
| e | 4.66 | 18.30 |

**Table S2.** The adsorption energy ($E_{ads}$, eV) and the cohesive energy ($E_{coh}$, eV) of the different transition metals doped on N-C nanosheets.

| **SAC** | $E_{ads}$ | $E_{ads} + E_{coh}$ |
|---|---|---|
| Cr | -6.84 | -2.74 |
| Fe | -7.76 | -3.48 |
| Ni | -7.93 | -3.49 |
| Cu | -5.22 | -1.73 |
| Zn | -3.60 | -2.25 |
| Mo | -6.37 | 0.45 |
| Rh | -7.60 | -1.85 |
| **BAC** | $E_{ads}$ | $E_{ads} + E_{coh}$ |

| | | |
|---|---|---|
| Cr | -15.55 | -7.35 |
| Fe | -16.51 | -7.95 |
| Ni | -13.10 | -4.22 |
| Cu | -11.24 | -4.26 |
| Zn | -6.79 | -4.09 |
| Mo | -18.32 | -4.68 |
| Rh | -16.61 | -5.11 |

**Table S3.** Calculated values of $E^{o}_{M^{z+}/M\text{-}N\text{-}C}$ (V).

| SAC | | | |
|---|---|---|---|
| metal | $E^{o}_{M^{z+}/M}$ | z | $E^{o}_{M^{z+}/M\text{-}N\text{-}C}$ |
| Cr | −0.74 | 3 | 0.173 |
| Fe | −0.44 | 2 | 1.299 |
| Ni | −0.25 | 2 | 1.494 |
| Cu | 0.337 | 2 | 1.202 |
| Zn | −0.7618 | 2 | 0.363 |
| Mo | no data | | |
| Rh | 0.76 | 3 | 1.376 |
| **BAC** | | | |
| **metal** | $E^{o}_{M^{z+}/M}$ | z | $E^{o}_{M^{z+}/M\text{-}N\text{-}C}$ |
| Cr | −0.74 | 3 | 1.709 |
| Fe | −0.44 | 2 | 3.533 |
| Ni | −0.25 | 2 | 1.859 |
| Cu | 0.337 | 2 | 2.466 |
| Zn | −0.7618 | 2 | 1.282 |
| Mo | no data | | |
| Rh | 0.76 | 3 | 2.463 |

**Table S4.** Adsorption energy (eV) of $N_2$, NNH, and $NH_2$ intermediates on the different N-C monolayers.

| | SAC | | | BAC | | |
|---|---|---|---|---|---|---|
| | $N_2$ | NNH | $NH_2$ | $N_2$ | NNH | $NH_2$ |
| Cr | -0.35 | 0.25 | -1.02 | 0.48 | 0.05 | -1.72 |
| Fe | -0.73 | 1.02 | -0.69 | -0.58 | -0.01 | -1.16 |
| Ni | -0.15 | 1.74 | 0.66 | -0.13 | 1.67 | 0.37 |
| Cu | -0.42 | 1.84 | 1.01 | -0.49 | 2.24 | 1.04 |
| Zn | -0.38 | 2.03 | 0.06 | -0.26 | 1.71 | -0.28 |
| Mo | -1.29 | -1.43 | -2.78 | -0.82 | -1.07 | -2.25 |
| Rh | -0.09 | 0.42 | -0.69 | -0.15 | 2.01 | 0.45 |

**Table S5.** Atomic configurations and corresponding adsorption energy and free energy correction of each elementary steps, along the different pathways for Mo-N-C.

| **Distal** | | | |
|---|---|---|---|
| Species | configurations | ΔE (eV) | ΔZPE − TΔS +∫CpdT (eV) |
| NN | 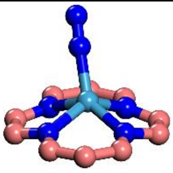 | -1.25 | 0.16 |
| NNH | 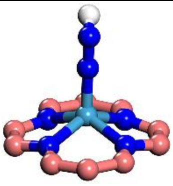 | -1.1 | 0.46 |
| $NNH_2$ | 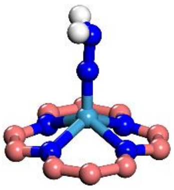 | -2.14 | 0.74 |
| N | 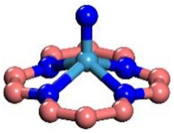 | -1.44 | 0.11 |

| | | | |
|---|---|---|---|
| NH | 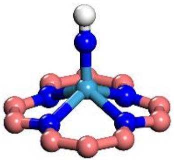 | -2.58 | 0.34 |
| $NH_2$ | 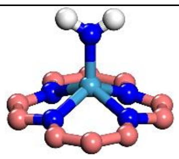 | -2.78 | 0.6 |

**Alternating**

| species | configurations | ΔE (eV) | $\Delta ZPE - T\Delta S + \int CpdT$ (eV) |
|---|---|---|---|
| NN | 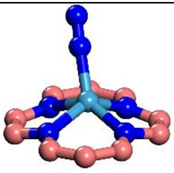 | -1.25 | 0.16 |
| NNH | 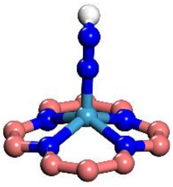 | -1.1 | 0.46 |
| NHNH | 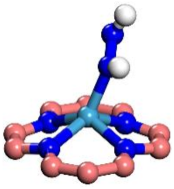 | -0.68 | 0.74 |
| $NHNH_2$ | 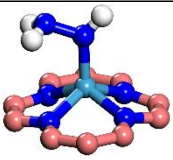 | -2.06 | 1.01 |
| $NH_2NH_2$ | 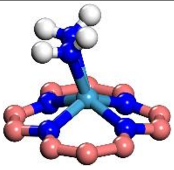 | -1.53 | 1.43 |
| $NH_2$ | 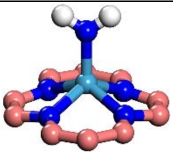 | -2.78 | 0.6 |

| Enzymatic | | | |
|---|---|---|---|
| species | configurations | ΔE (eV) | $\Delta ZPE - T\Delta S + \int C_p dT$ (eV) |
| NN | 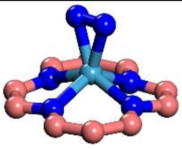 | -1.29 | 0.19 |
| NNH | 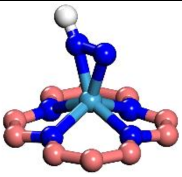 | -1.43 | 0.45 |
| NHNH | 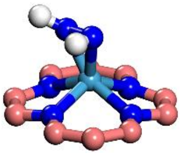 | -1.5 | 0.74 |
| $NHNH_2$ | 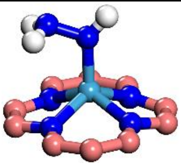 | -2.37 | 1.08 |
| $NH_2NH_2$ | 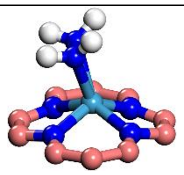 | -1.7 | 1.42 |
| $NH_2$ | 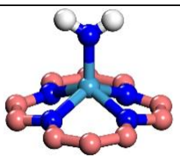 | -2.78 | 0.6 |

**Table S6.** Atomic configurations and corresponding adsorption energy and free energy correction of each elementary steps, along the different pathways for $Mo_2$-N-C.

| **Distal** | | | |
|---|---|---|---|
| species | configurations | ΔE (eV) | ΔZPE − TΔS +∫CpdT (eV) |
| NN | 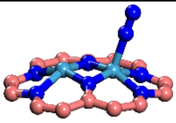 | -0.82 | 0.11 |
| NNH | 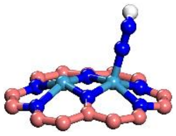 | -0.6 | 0.42 |
| $NNH_2$ | 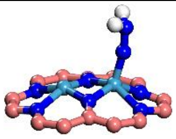 | -1.11 | 0.72 |
| N | 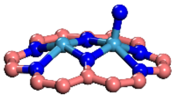 | -0.31 | 0.05 |
| NH | 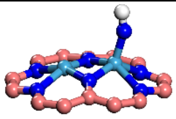 | -1.41 | 0.32 |
| $NH_2$ | 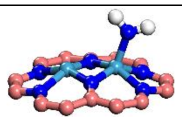 | -2.25 | 0.64 |
| **Alternating** | | | |
| species | configurations | ΔE (eV) | ΔZPE − TΔS +∫CpdT (eV) |
| NN | 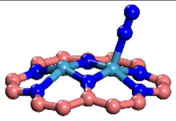 | -0.82 | 0.11 |
| NNH | 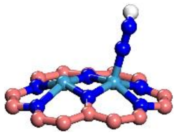 | -0.60 | 0.42 |

| | | | |
|---|---|---|---|
| NHNH | 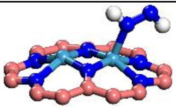 | -0.54 | 0.73 |
| NHN$H_2$ | 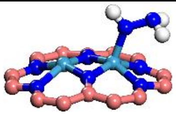 | -1.67 | 1.08 |
| $NH_2NH_2$ | 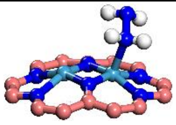 | -1.61 | 1.38 |
| $NH_2$ | 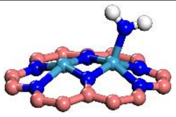 | -2.25 | 0.64 |

**Enzymatic**

| species | configurations | ΔE (eV) | ΔZPE − TΔS +∫CpdT (eV) |
|---|---|---|---|
| NN | 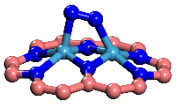 | -0.32 | 0.19 |
| NNH | 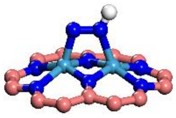 | -1.07 | 0.53 |
| NHNH | 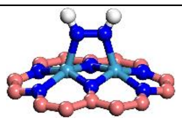 | -1.92 | 0.85 |
| NHN$H_2$ | 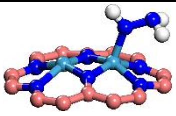 | -1.59 | 1.13 |
| $NH_2NH_2$ | 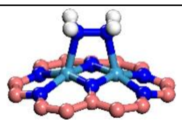 | -0.98 | 1.34 |
| $NH_2$ | 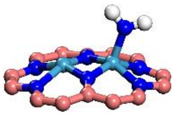 | -2.25 | 0.64 |

**Table S7.** Different two-dimensional NRR electrocatalysts reported in the literature.

| Electrocatalysts | $\Delta G_{[*NH2-NH3(g)]}$ (eV) | References |
|---|---|---|
| Mo-B-N | ~ 0.98 | *J. Am. Chem. Soc.* **2017,** *139,* 12480-12487. |
| $BC_3$ | ~ 2.13 | *Joule* **2018,** *2,* 1–13 |
| NPC-pyridinic N | ~ -0.15 | *ACS Catal.* **2018,** *8,* 1186-1191. |
| $B_{int}$-doped $C_2N$ | 3.03 | *J. Mater. Chem. A* **2019,** *7,* 2392-2399. |
| Ru/B$\alpha$ | 0.26 | *J. Mater. Chem. A* **2019,** *7,* 4771-4776. |
| Boron Antisites of BNNT | 1.53 | *Phys. Chem. Chem. Phys.* **2017,** *19,* 15377-15387. |
| $Mo_2$-N-C | 1.05 | This work |
